# Anti-Inflammatory Activity of *Choisya ternata* Kunth Essential Oil, Ternanthranin, and Its Two Synthetic Analogs (Methyl and Propyl *N*-Methylanthranilates)

**DOI:** 10.1371/journal.pone.0121063

**Published:** 2015-03-25

**Authors:** Mariana Martins Gomes Pinheiro, Ana B. Miltojević, Niko S. Radulović, Ikarastika Rahayu Abdul-Wahab, Fabio Boylan, Patrícia Dias Fernandes

**Affiliations:** 1 Universidade Federal do Rio de Janeiro, Instituto de Ciências Biomédicas, Laboratório de Farmacologia da Dor e da Inflamação, Rio de Janeiro, Brasil; 2 Department of Chemistry, Faculty of Science and Mathematics, University of Niš, Višegradska 33, 18000 Niš, Serbia; 3 School of Pharmacy and Pharmaceutical Sciences, Trinity Biomedical Sciences Institute, Trinity College Dublin, Dublin 2, Ireland; Federal University of Rio de Janeiro, BRAZIL

## Abstract

*Choisya ternata* Kunth (Rutaceae) is native to North America where it is popularly known as “Mexican orange”. In this study, the anti-inflammatory effects of the essential oil (EO) obtained from the leaves of *C*. *ternata*, one of its minor components (ternanthranin—ISOAN) and its two synthetic analogues (methyl and propyl *N*-methylanthranilate – MAN and PAN) were evaluated. Mice pretreated with the EO (EO) obtained from *C*. *ternata* leaves (3–100 mg/kg, p.o.), ISOAN, MAN or PAN (1–30 mg/kg, p.o.) and the reference drugs, morphine (1 mg/kg, p.o.) and acetylsalicylic acid (ASA, 100 mg/kg, p.o.), were evaluated in inflammation models such as formalin and subcutaneous air pouch models, with measurement of cell migration, exudate volume, protein extravasation, nitric oxide and pro-inflammatory cytokines. The EO from *C*. *ternata* significantly inhibited the time that the animals spent licking the formalin-injected paw in the second phase of the model at their higher doses (30 and 100 mg/kg, respectively). An inhibition of the inflammatory reaction induced after subcutaneous carrageenan injection into air pouch was also observed. In this model, the EO significantly reduced cell migration, exudate volume, protein extravased, and the increase in levels of inflammatory mediators (nitric oxide, TNF-α and IL-1β). ISOAN, MAN and PAN behaved in the same fashion at much smaller doses. Also, these molecules were able to show significant effects in the reduction of paw edema (at all tested doses) when the phlogistic agent was carrageenan, bradykinin, 5-HT, PGE2, C48/80 or 12-O-tetradecanoylphorbol-acetate (TPA). None of the tested doses had any effect in reducing histamine-induced edema. Our results indicate that the EO from *C*. *ternata* and anthranilate derivatives demonstrates an anti-inflammatory effect.

## Introduction

Inflammation is the body’s response to tissue injury, infection or invasion by microorganisms and its purpose is to keep homeostasis at normal. The molecular responses to inflammation are increasingly well understood. The inflammatory process is characterized by cellular and microvascular reactions that serve to remove damaged and generate new tissue. During the inflammatory process there is an activation of the innate immunity. Also, there is an increased production of various mediators including pro-inflammatory cytokines and eicosanoids [1, 2]. However, an excessive response can be associated to diseases with a significant inflammatory component being the case of asthma, rheumatoid arthritis, gout, autoimmune diseases and heart ischemia [3, 4].

The conventional anti-inflammatory drugs used to relieve the inflammatory process are either too expensive or with several side effects (i.e., hemorrhage and gastric ulcers). In this sense, pharmaceutical companies have strongly been supporting the search and development of drugs with anti-inflammatory activity from medicinal plants.

Natural products continue to play an important role in human history, for the treatment of a variety of inflammatory diseases [5]. Currently, most of the drugs in clinical use are of natural origin or were developed by chemical synthesis planned from natural products [6].

*C*. *ternata* Kunth (Rutaceae) popularly known as “Mexican orange” and “Hierba del Clavo” is native to North America, being cultivated in the central and southern mountains of Mexico [7, 8]. There are previous reports of this herb’s traditional use in Mexico [7, 8]. Previous phytochemical studies of this plant have shown the presence of quinolone alkaloids, among other compounds [7, 9, 10–13]. Recently, our research group has isolated a new compound from the hexane extract of *C*. *ternata* leaves, choisyaternatine, together with the known compounds tecleamaniensine A, lup-20(29)-en-3β-ol (lupeol), lup-20(29)-en-3β,24-diol, β-sitosterol glucoside and skimmianine [14]. We also demonstrated the identification of a new alkaloid (isopropyl *N*-methylanthranilate, named ternanthranin) obtained from the EO of *C*. *ternata* Kunth. Ternanthranin (here called ISOAN) was synthesized together with its two analogues methyl (MAN) and propyl *N*-methylanthranilate (PAN) [15], the three of them with antinociceptive effects [16]. Additionally, also recently, ISOAN and MAN were shown to possess anxiolytic and antidepressant properties [17], as well as gastro- and hepatoprotective activities [18, 19]. In this work we aimed to investigate the anti-inflammatory effect of the EO from *C*. *ternata* leaves as well as ISOAN, MAN and PAN using *in vivo* and *in vitro* models of inflammation. Moreover, attempts have also been done to analyze some of the possible mechanisms of action for these anthranilate derivatives.

## Materials and Methods

### Plant material

Leaves from *C*. *ternata* Kunth were collected in October 2008 in the Trinity College Botanical Gardens, Dublin (living plant accession number 19850023). A voucher sample was deposited in the Herbarium of Trinity College Dublin under the collection number SW 10–52.

### Extraction of the essential oil and the identification of its constituents

The material was dried at room temperature for one week. A portion (250 g) was submitted to hydrodistillation with 2.5 l of distilled water for 2.5 h using a Clevenger-type apparatus yielding the EO (0.10%, v/w). The obtained oil was separated by extraction with diethyl ether (Merck, Germany), dried over anhydrous magnesium sulfate (Aldrich, USA). Another portion (1 g) was macerated for three days with ethanol (100 ml). The obtained extract was evaporated to dryness *in vacuo* at room temperature. The identification of *C*. *ternata* EO and solvent extract constituents has been described before [14, 15]. The EO of *C*. *ternata* was used for further assays.

### Synthesis of ternanthranin, methyl and propyl *N*-methylanthranilate

Ternanthranin (isopropyl *N*-methylanthranilate, ISOAN), methyl *N*-methylanthranilate (MAN) and propyl *N*-methylanthranilate (PAN) were synthesized as described in [15] and characterized by infrared spectroscopy, mass spectrometry, and nuclear magnetic resonance (^1^H- and ^13^C-NMR).

### Animals

Male Webster mice (18–25 g) donated by Instituto Vital Brazil were used in this study. Animals were housed in a room with controlled temperature 22 ± 2°C for a 12 h light/dark cycle with free access to food and water. Twelve hours before each experiment animals received only water, in order to avoid food interference with substances absorption. Animals were acclimatized to the laboratory for at least 1 h before testing and were used only once throughout the experiments. After assays animals were euthanized with an overdose of choral hydrate. This study was carried out in strict accordance with the recommendations in the Guide for the Care and Use of Laboratory Animals of the adapted by Brazilian College of Animal Experimentation (COBEA), approved by the Biomedical Science Institute/UFRJ, Ethical Committee for Animal Research and received the number DFBCICB-015.

### Drugs and treatment

Acetylsalicylic acid (ASA), dexamethasone, carrageenan, bradykinin, serotonin, prostaglandin E2 (PGE2), 12-*O*-tetradecanoylphorbol-acetate (TPA) and histamine were purchased from Sigma Aldrich (St. Louis, MO, U.S.A.), formalin was from Merck (Germany). Morphine sulfate was kindly donated by Cristália (São Paulo, Brazil). Dexamethasone was purchased from Aché (Brazil).

All drugs were diluted in phosphate buffer saline (PBS) just before use. EO, ternanthranin (ISOAN), methyl- (MAN) and propyl *N*-methylanthranilate (PAN) were dissolved in dimethyl sulfoxide (DMSO) to prepare a stock solution (100 mg/ml). On the day of the experimental protocol, the stock solution was diluted with PBS. The animals were treated by oral gavage 60 min prior to experiments with EO at doses of 3 to 30 mg/kg; or with ISOAN, MAN or PAN at doses of 1 to 10 mg/kg, in a final volume of 0.1 ml per animal. For the paw edema model, the animals received the PBS and ISOAN, MAN or PAN in the concentration of 10 nmol/paw in a final volume of 50 μl 15 min before intraplantar injection of different inflammatory mediators.

Morphine (1 mg/kg, p.o.), ASA (100 mg/kg, p.o.) and dexamethasone (5 mg/kg, i.p.) were used as reference drugs. The control group was composed of vehicle treated animals (PBS with the same amount of the highest concentration of DMSO used). The final concentration of DMSO did not exceed 0.5% (v/v) at which had no effect *per se*.

### Formalin test

In order to provide more evidence regarding the EO effects, the licking behavior was examined immediately after formalin hind paw injection. The procedure was similar to the method described by [20]. Animals received 20 μl of formalin (2.5%, v/v) into the dorsal surface of the left hind paw. Animals were individually observed and the time that they spent licking the formalin-injected paw recorded. The nociceptive response develops in two phases: the first 5 min after formalin injection (first phase, neurogenic pain response); the second 15–30 min after the formalin injection (second phase, inflammatory pain response).

### Subcutaneous air pouch (SAP) model

The experimental protocol was similar to that described by [21] with several modifications described in [22]. The animals received a dorsal subcutaneous injection of sterile air (10 ml) on three alternate days to induce the SAP. On the sixth day, animals received a subcutaneous injection of sterile carrageenan suspension (1%; 1 ml). Mice were pretreated with vehicle, EO (3, 10 or 30 mg/kg, p.o.), ISOAN, MAN or PAN (1, 3 or 10 mg/kg, p.o.) or dexamethasone (5 mg/kg, i.p.) 1 h before carrageenan injection into the SAP. The control group received an injection of sterile PBS (1 ml) into the SAP. Animals were sacrificed 24 h after carrageenan injection. The cavity was washed with 1 ml of sterile PBS. Exudates were collected and quantified. The total cells counts were determined in the exudates using a CellPocH-100iV Diff (Sysmex) hematology analyzer. The exudates were centrifuged at 11,000 rpm for 10 min at 4°C and aliquots of the supernatants were stored at −20°C until the dosages.

### Quantification of TNF-α, IL-1β and protein

Supernatants from the exudate collected in the SAP were used to measure tumor necrosis factor-α (TNF-α), interleukin-1β (IL-1β) and extravased protein. TNF-α and IL-1β quantifications were done by enzyme-linked immunosorbent assay (ELISA) according to the manufacturer’s instructions (B&D, USA). The results are expressed as pg/ml of each cytokine. The extravased proteins were determined using the BCA method (BCA™ Protein Assay Kit, Pierce) following the instructions' protocol.

### Paw edema

The method used was similar to the one described by Ferreira [23]. Carrageenan (300 μg), histamine (9 μmol), bradykinin (3 nmol), PGE2 (3 nmol), serotonin (2.3 nmol), TPA (50 pmol) were injected in the mice paw 15 min after intraplantar injection of ISOAN, MAN or PAN (10 nmol) or PBS. Paw edema was measured by pletismography several times after phlogistic agent’s injections. The results were converted to edema (in μl).

### Cell culture

RAW 264.7 mouse monocyte-macrophages (ATCC TIB-71) were grown in plastic bottles in a RPMI 1640 medium supplemented with 10% fetal bovine serum, glutamine (2 mM) and HEPES (15 mM) (from now named RPMI) in a humidified atmosphere containing 5% CO_2_ and 95% air at 37°C. When cultures formed a confluent monolayer cells were trypsinised, centrifuged and put to adhere in 96 or 12 wells plate with RPMI at a density of 2 x 10^6^ cell/ml.

### Cytotoxicity assay by MTT

The mitochondrial-dependent reduction of 3-(4,5-dimethylthiazol-2-yl)-2,5-diphenyltetrazolium bromide (MTT) to formazan was used to measure cell respiration as an indicator of cell viability [24]. Briefly, after 24 hours incubation of RAW 264.7 adherent cells with or without ISOAN, MAN or PAN (10, 30 or 100 μM), supernatants were changed by 100 μl of RPMI medium containing 0.5 mg/ml MTT and cells incubated for 1 hour at 37°C in a 5% CO_2_ atmosphere. After the aspiration of the medium, 100 μl of DMSO was added to the cells to dissolve the formazan. The absorbance from each group was measured in a FlexStation microplate reader (Molecular Devices, USA) at 540 nm. The control groups consisted of cells with medium plus vehicle used to dissolve anthranilates and was considered as 100% of viable cells. Results are expressed as percentage of viable cells when compared to the control groups.

### Nitrate and nitrite measurement

To evaluate NO production in cell culture, nitrite concentration in the supernatants of RAW 264.7 adherent cells was measured using the Griess reaction [25]. Cells were activated with LPS (1 μg/ml) and further incubated with ISOAN, MAN or PAN (10, 30 or 100 μM). After 24 h incubation, 100 μl of the supernatant was obtained to measure nitrite. To evaluate NO production in SAP exudate, nitrate (the stable metabolite of NO) concentrations were measured according to the protocol adapted by [22].

The nitrite concentrations (obtained after nitrate conversion or from cell culture supernatant) were measured after mixtures of equal parts of sample and Griess Reagent (1% sulphanilamide, 0.1% naphthylethylene diamine dihydrochloride, 10% H_3_PO_4_) and incubation for 10 min at room temperature [25]. The absorbance was read at 540 nm in a FlexStation microplate reader (Molecular Devices, USA) and the nitrite concentration was calculated using a standard curve of sodium nitrite.

### Nitric oxide-trapping capacity of anthranilates

To test the capacity of anthranilates in trapping nitric oxide, we used a cell-free system as described in [26]. SNAP (*S*-nitroso-*N*-acetyl DL-penicillamine) was used. When in solution SNAP liberates to the medium nitric oxide which is transformed to nitrite [27]. The addition of a NO scavenger to the SNAP solution results in a decay in the supernatant nitrite accumulation. Using this protocol, each anthranilate (in concentration of 100 μM) was incubated with 1 mM of SNAP. After 6 h incubation, an aliquot of supernatant was removed and quantified by Griess reaction [25]. Results are expressed as μM of nitrite calculated in comparison with the sodium nitrite standard curve.

### Statistical analysis

Experimental groups consisted of 6–10 mice for all *in vivo* assays. A total of 348 mice were used throughout the study.For the *in vitro* assays, all groups were done in triplicate and each protocol was repeated at least 4 times. The results are presented as mean ± S.D. Statistical significance between groups was evaluated by applying analyses of variance (ANOVA) followed by Bonferroni’s test. *P* values less than 0.05 (*p < 0*.*05*) were considered significant.

## Results

### Effect of the essential oil from *C*. *ternata* in the formalin-induced licking test

**Fig 1:**
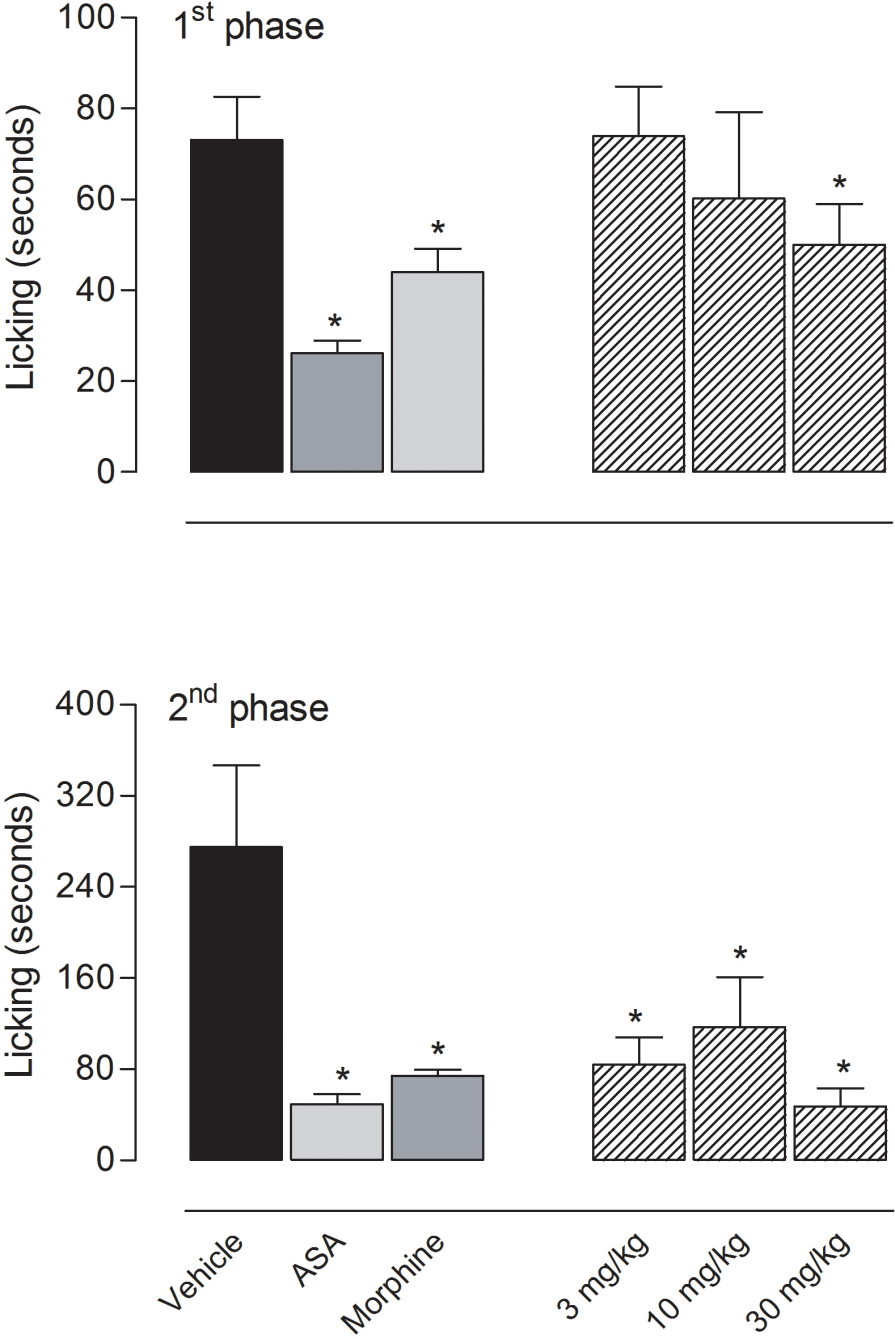
Effects of the essential oil (EO) from *Choisya ternata* Kunth leaves in the first and second phase of the formalin-induced licking in mice. Animals were pretreated by oral administration with different doses of the EO, acetylsalicylic acid (ASA, 100 mg/kg), morphine (1 mg/kg), or vehicle. Results were presented as mean ± S.D. (n = 6–10) of the time that the animal spent licking the formalin-injected paw. Statistical significance was calculated by ANOVA followed by Bonferroni's test. *p<0.05 when compared to the vehicle-treated group.

Previous results from our group demonstrated that the essential oil (EO) from *C*. *ternata* leaves significantly inhibited acetic acid-induced contortions [15]. Considering these results, we decided to evaluate the anti-inflammatory effects of EO in the formalin test. The intraplantar injection of formalin resulted in a biphasic response. In the first phase (neurogenic phase), the response was immediate and lasted up to 5 min following formalin injection resulting in the licking time of the 73 ± 9.5 seconds, whereas the second phase (inflammatory phase) began at 15 min and lasted up to 30 min after formalin injection and resulted in 275 ± 72 seconds in the vehicle-treated group. Fig. 1 shows that the pretreatment with EO produced a significant inhibition in both phases. In the first phase, the highest dose (30 mg/kg) produced an antinociceptive effect with 35.4% reduction in the licking time (47.2 ± 16 seconds *versus* 73 ± 9.5 seconds in the vehicle-treated group). The results obtained in the second phase show that the administration of EO in the dose of 10 mg/kg (117 ± 44 seconds; 57.4% reduction) and 30 mg/kg (84 ± 24 seconds; 69.4% reduction) significantly reduced the licking response to the formalin injection. EO at the doses of 30 mg/kg had an effect similar to that obtained with ASA (100 mg/kg), an non-steroidal anti-inflammatory drug, that reduced formalin-licking response in 78% in the second phase (49 ± 8.4 seconds in ASA-treated group *versus* 275 ± 72 seconds in the vehicle-treated group) (Fig. 1).

### Effect of EO from *C*. *ternata* on cell migration into the subcutaneous air pouch

The inhibitory effects caused by EO-treatment in the second phase of the formalin model (inflammatory phase) led us to evaluate its effects in another model of inflammation, the subcutaneous air pouch (SAP) model. A subcutaneous injection of sterile air induced formation of a structure similar to the synovium. In the SAP model, the injection of carrageenan induces an inflammatory process characterized by cell migration, formation of exudate and production of inflammatory mediators accumulated in the cavity.

**Fig 2:**
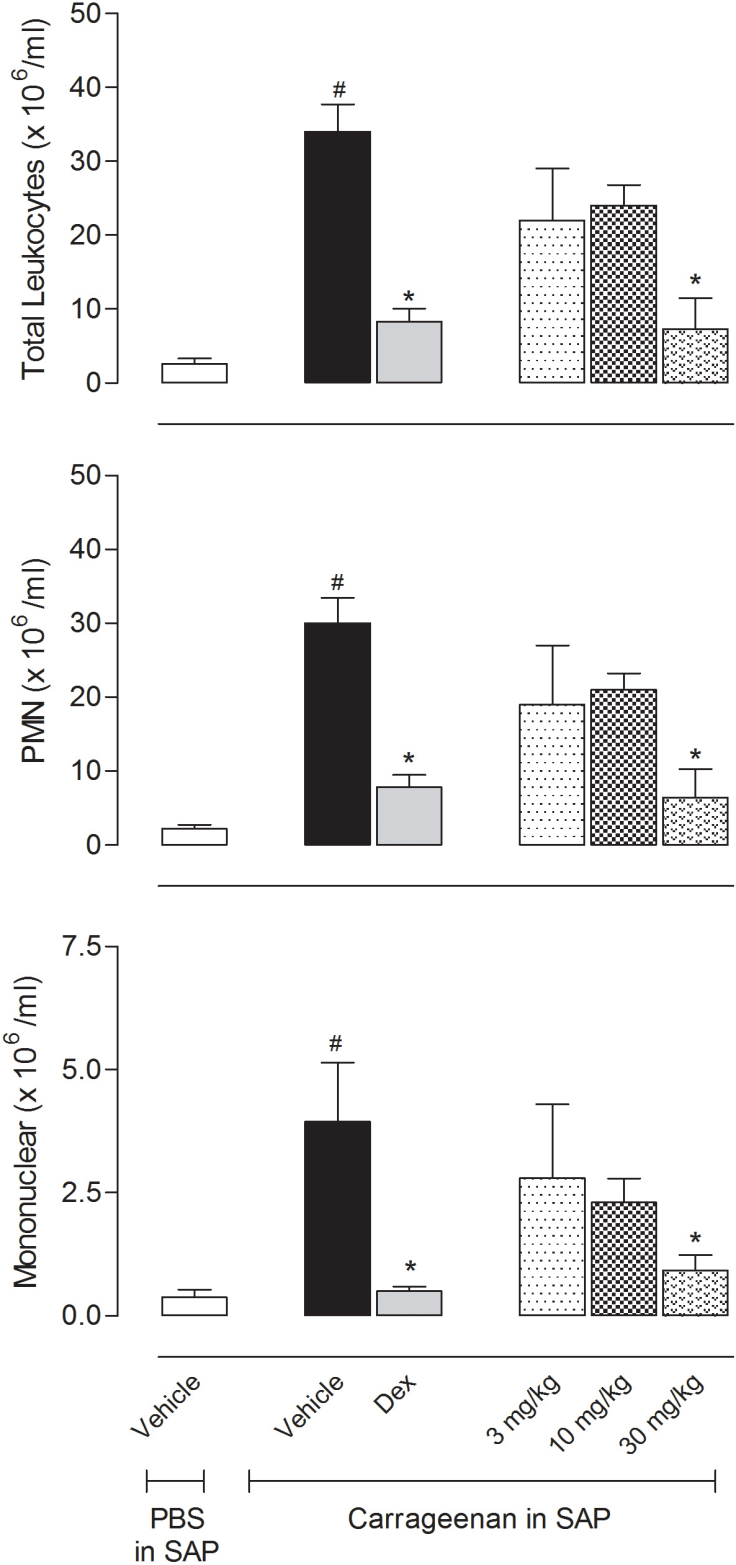
Effects of the essential oil (EO) from *Choisya ternata* Kunth leaves on the total number of leukocyte, polymorphonuclear and mononuclear cells in the subcutaneous air pouch (SAP). Animals were pretreated with different doses of EO, dexamethasone (Dex, 5 mg/kg, i.p.) or vehicle 1 h prior to carrageenan (1%) injection into the SAP. Results were presented as mean ± S.D. (n = 6–10) of cells (x 10^6^/ml) in the SAP. Statistical significance was calculated by ANOVA followed by Bonferroni's test. ^#^ p<0.05 when compared to the vehicle-treated animals in the group of PBS in SAP; *p<0.05 compared to the vehicle-treated animals in the group of carrageenan in the SAP.

Injection of 1 ml of carrageenan (1%) into the SAP markedly increased the exudate volume and resulted in a 13-fold increase in the number of leukocytes in the vehicle-treated group (34 ± 3.7 x 10^6^ cells/ml in the carrageenan-injected group *versus* 2.6 ± 0.8 x 10^6^ cells/ml in the vehicle-injected group). The differential cell count of the exudate from animals receiving an injection of carrageenan into the SAP was evidenced in an increase in polymorphonuclear (30.2 ± 2.7 x 10^6^ cells/ml) and mononuclear cells (3.8 ± 1.2 x 10^6^ cells/ml) when compared with PBS group (2.2 ± 0.4 x 10^6^ cells/ml and 0.38 ± 0.16 x 10^6^ cells/ml, respectively). When mice were pretreated with the steroidal anti-inflammatory drug, dexamethasone (5 mg/kg, i.p.), a significant reduction in the leukocyte migration in almost 76% (34 ± 3.7 x 10^6^ cells/ml in the carrageenan-injected group *versus* 8.3 ± 1.7 x 10^6^/ml in the dexamethasone-pretreated group) was observed. A proportional decrease in the number of polymorphonuclear and mononuclear cells was also observed (Fig. 2).

Pretreatment with EO (at 30 mg/kg) was effective in reducing the number of leukocytes that migrated to the SAP, with inhibition of 78.5% (7.3 ± 4.2 x 10^6^ cells/ml). EO reduced in 78.8% (6.4 ± 3 x 10^6^ cells/ml) the number of polymorphonuclear cells in the SAP at the dose of 30 mg/kg. A reduction in mononuclear cells that migrated to the cavity, 76.4% (0.9 ± 0.3 x 10^6^ cells/ml) could also be observed (Fig. 2).

### Effects of EO from *C*. *ternata* on protein extravasation, nitric oxide and cytokine production

**Fig 3:**
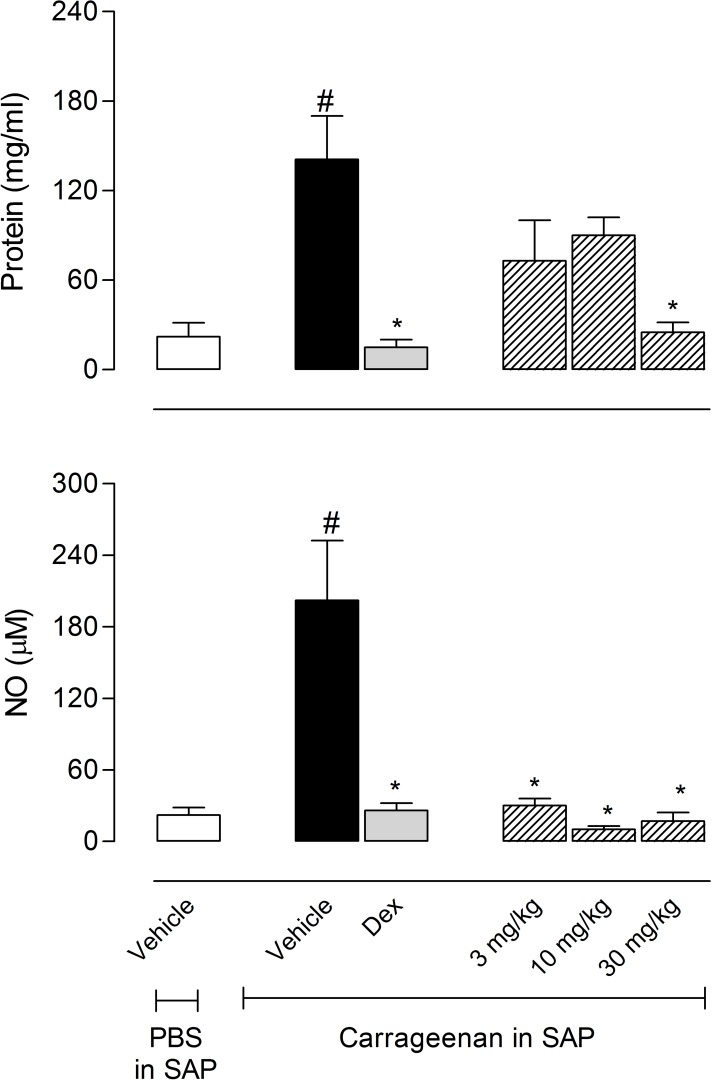
Effects of the essential oil (EO) from *Choisya ternata* Kunth leaves on protein extravasation or nitric oxide (NO) production in the subcutaneous air pouch (SAP) model. Animals were pretreated with different doses of EO, dexamethasone (Dex, 5 mg/kg, i.p.) or vehicle 1 h prior to carrageenan (1%) injection into the SAP. Results were presented as mean ± S.D. (n = 6–8) of protein (mg/ml) or NO levels (μM). Statistical significance was calculated by ANOVA followed by Bonferroni's test. ^#^p<0.05 when compared to vehicle-treated animals in the group of PBS in SAP; *p<0.05 compared to the vehicle-treated animals in the group of carrageenan in the SAP.

One can see from Fig. 3 that the injection of carrageenan produced a 6.4-fold increase in protein accumulated in the SAP (22 ± 9.5 mg/ml for the group that received PBS in the SAP *versus* 141 ± 29 mg/ml for the group that received carrageenan in the SAP). The pretreatment of mice with the highest doses of EO (30 mg/kg) significantly reduced the carrageenan-induced protein leakage by 82.3%.

Carrageenan also drastically increased the levels of nitric oxide (NO, 202 ± 50 μM) in the SAP exudate when compared with the group that received PBS (22 ± 6.5 μM). Interestingly, the results demonstrated that the pretreatment of mice with all doses of EO significantly inhibited the production of NO in the SAP. The lowest dose inhibited 85.1% and the highest dose inhibited 92% of NO production (Fig. 3).

**Fig 4:**
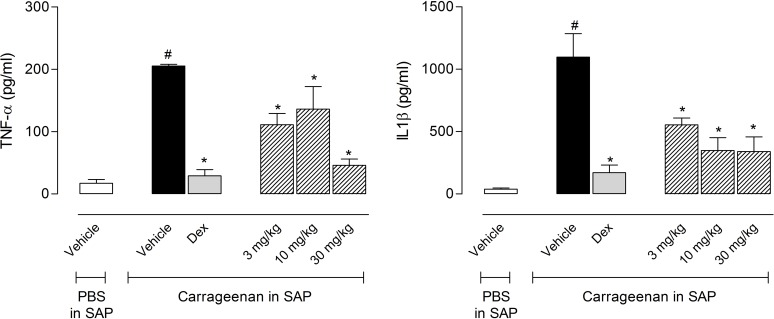
Effects of the essential oil (EO) from *Choisya ternata* Kunth leaves on TNF-α and IL-1β production in the subcutaneous air pouch (SAP) model. Animals were pretreated with different doses of EO, dexamethasone (Dex, 5 mg/kg, *i*.*p*.) or vehicle 1 h prior to carrageenan (1%) injection into the SAP. Results were presented as mean ± S.D. (n = 6–8) of TNF-α (pg/mL) or IL-1β (pg/ml). Statistical significance was calculated by ANOVA followed by Bonferroni's test. ^#^p<0.05 when compared to the vehicle-treated animals in the PBS group; *p<0.05 compared to the vehicle-treated animals in the carrageenan group.

Levels of TNF-α increased twelve times after carrageenan injection in the SAP (17 ± 6.2 pg/ml in the vehicle-treated group *versus* 205 ± 3 pg/ml in the carrageenan-injected group). All tested doses of EO significantly reduced the levels of this cytokine. Similarly, carrageenan caused a 38-fold increase of the levels of IL-1β (38 ± 10 pg/ml for the vehicle-treated group *versus* 1,096 ± 190 pg/ml for the carrageenan-injected group). All doses of EO significantly reduced the levels of IL-1β (Fig. 4).

As the EO from *C*. *ternata* demonstrated a significant effect in models of inflammation (*i*.*e*., formalin-induced licking and migration into subcutaneous air pouch), we decided to evaluate a possible anti-inflammatory effect of isopropyl *N*-methylanthranilate (ternanthranin—ISOAN), a new compound identified in the EO of *C*. *ternata*, and its two analogs, methyl *N*-methylanthranilate (MAN), and propyl *N*-methylanthranilate (PAN) obtained by synthesis.

**Fig 5:**
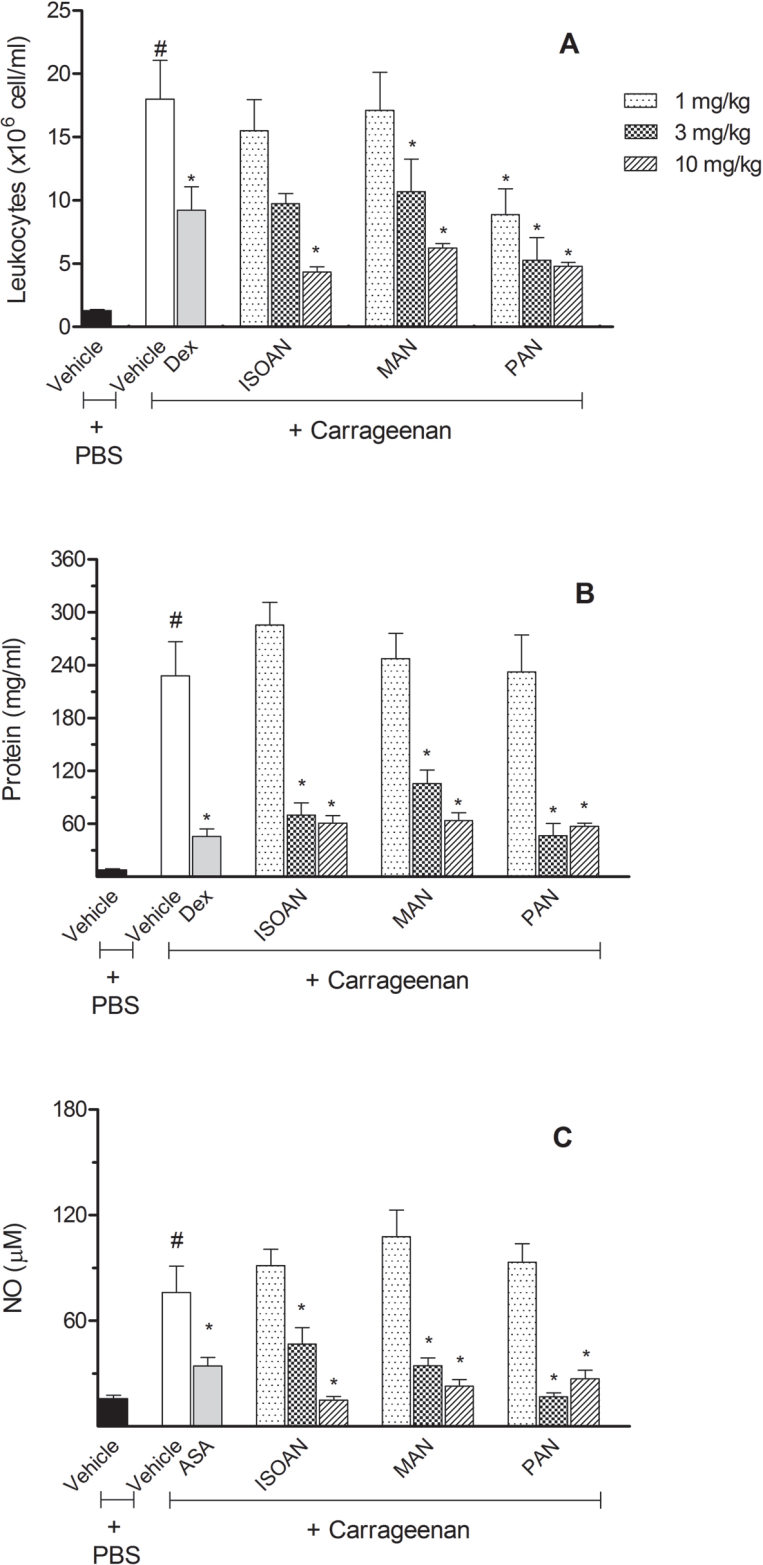
Effects of ternanthranin (ISOAN), MAN and PAN on total leukocyte count, amount of extravased protein or nitric oxide (NO) produced in the subcutaneous air pouch (SAP). Animals were pretreated with different doses of ISOAN, MAN or PAN, dexamethasone (Dex, 5 mg/kg, i.p.) or vehicle 1 h prior to carrageenan (1%) injection into the SAP. Results were presented as mean ± S.D. (n = 6–10) of cells (x 10^6^/ml) or protein (mg/ml) extravased to the SAP. Statistical significance was calculated by ANOVA followed by Bonferroni's test. ^#^p<0.05 when compared to vehicle-treated animals in the group of PBS in SAP; *p<0.05 compared to the vehicle-treated animals in the group of carrageenan in the SAP.

In this way, the effects of these anthranilates were also evaluated on the SAP model. When the experimental animals were pretreated with MAN (doses of 1, 3 or 10 mg/kg), a significantly reduction in cell count was observed in the highest doses used. ISOAN demonstrated a significant reduction of cell migration at 10 mg/kg doses. Differently, PAN was the most effective in reducing leukocyte migration. All doses tested significantly reduced the number of cells that migrated to the SAP with 50.6%, 70.7%, and 73.4% of reduction corresponding to 1, 3, and 10 mg/kg, respectively (Fig. 5A).

Another parameter evaluated in the SAP model was the amount of proteins in the exudates. Fig. 5B shows that all three anthranilates (at 3 and 10 mg/kg) reduced the amount of extravased proteins. It was also observed that 3 and 10 mg/kg doses of all three anthranilates significantly reduced nitric oxide (NO) produced by cells (Fig. 5C).

**Fig 6:**
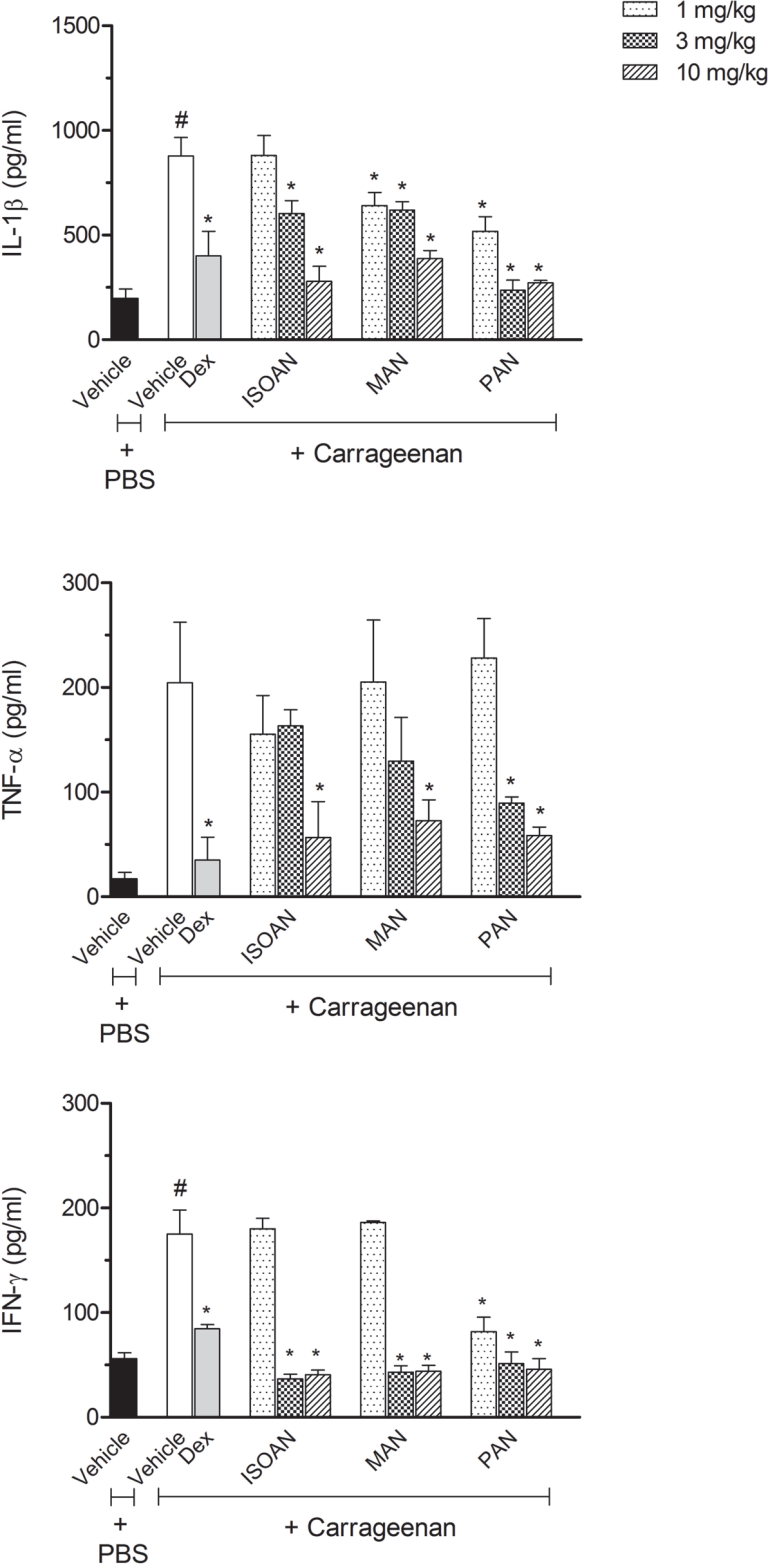
Effects of ternanthranin (ISOAN), MAN or ISOAN on cytokine production in the subcutaneous air pouch (SAP) model. Animals were pretreated with different doses of the ISOAN, MAN or PAN, dexamethasone (Dex, 5 mg/kg, i.p.) or vehicle 1 h prior to carrageenan (1%) injection into the SAP. Results were presented as mean ± S.D. (n = 6–8) of cytokine. Statistical significance was calculated by ANOVA followed by Bonferroni's test. ^#^p<0.05 when compared to the vehicle-treated animals in the PBS group; *p<0.05 compared to the vehicle-treated animals in the carrageenan group.

As shown in Fig. 6, for the carrageenan group, the cytokines (TNF-α, IFN-γ or IL-1β) extravased was significantly higher than for the vehicle group. Treatment with MAN and PAN (1, 3 and 10 mg/kg) significantly reduced IL-1β levels, while ISOAN only demonstrated a significant reduction at the doses of 3 and 10 mg/kg.

The amount of TNF-α was reduced only when animals were pretreated with 10 mg/kg of MAN and ISOAN or 3 and 10 mg/kg of PAN. The levels of IFN-γ were drastically reduced by the pretreatment with all anthranilates (in the doses of 3 and 10 mg/kg) (Fig. 6).

### Effect of anthranilates on paw edema

**Fig 7:**
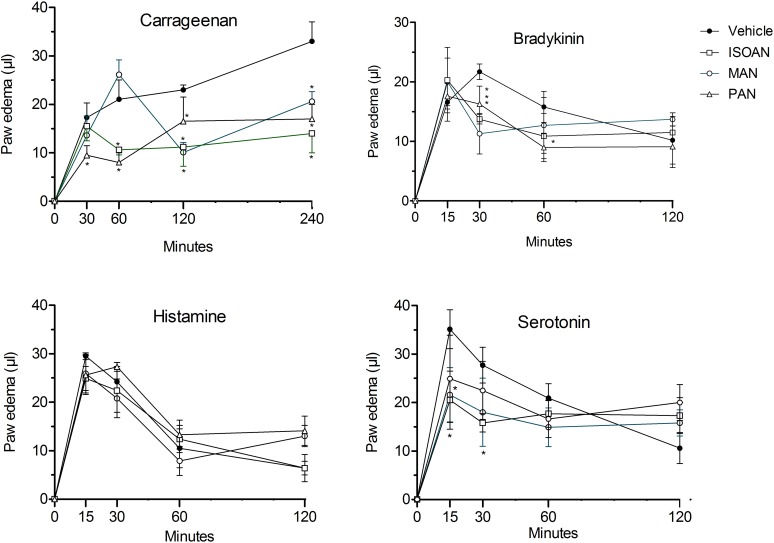
Effect of isopropyl-*N*-methylanthranilate (ternanthranin, ISOAN), MAN and PAN on mice paw edema induced by carrageenan (300 μg/paw), bradykinin (3 nmol/paw), histamine (9 μmol/paw) or serotonin (2.3 nmol/paw). Paw oedema was measured at several intervals after injection. Results were presented as mean ± S.D. (n = 6–8) of paw oedema (in μl). Statistical significance was calculated by ANOVA followed by Bonferroni's test. ^#^p<0.05 when compared to the vehicle-treated animals.

The *in vivo* anti-inflammatory effects of ISOAN, MAN and PAN were evaluated in the model of mice paw edema induced by carrageenan. This activity was demonstrated by pre-treating the animals with the compounds by their direct injection in the paws of mice to discard the possibility of the activity being produced by the liver metabolites of these compounds. Fig. 7 shows that the pretreatment of animals with ISOAN (ternanthranin) (10 nmol/paw) demonstrated effect during 1^st^, 2^nd^ and 4^th^ hour of the edema. MAN (10 nmol/paw) also significantly reduced the late phases (2 and 4 hours) of carrageenan-induced edema. Conversely, the same dose of PAN reduced all phases of carrageenan edema.

In order to further evaluate the effects of anthranilates, the edema was induced by specific phlogistic agents. In this way, bradykinin (BK, 3 nmol), histamine (9 μmol) or serotonin (5HT, 2.3 nmol) were injected in mice paws. The BK-, 5HT- or histamine-induced paw swelling were maximal 15 min after the injection and reduced thereafter. As can be seen from Fig. 7, all three anthranilates (10 nmol/paw), locally injected, significantly reduced BK- and 5HT-induced edema. However, none of them significantly reduced histamine-induced edema (Fig. 7).

**Fig 8:**
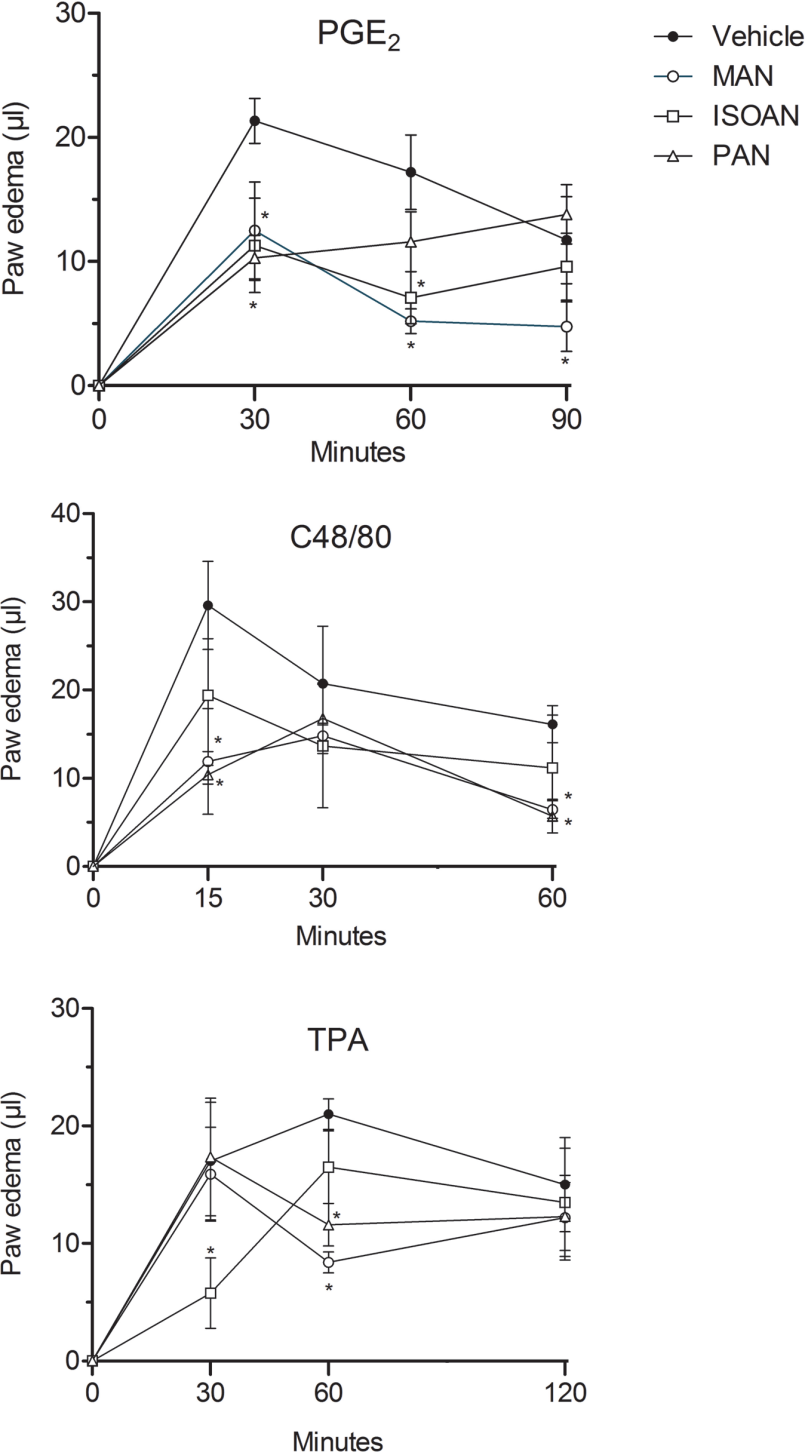
Effect of ternanthranin (ISOAN), MAN and PAN on mice paw oedema induced by prostaglandin E2 (PGE2, 3 nmol/paw), compound 48/80 (100 nmol/paw) or TPA (50 pmol/paw). Paw oedema was measured at several intervals after the injections. Results are presented as mean ± S.D. (n = 6–8) of paw edema (in μl). Statistical significance was calculated by ANOVA followed by Bonferroni's test. ^#^p<0.05 when compared to the vehicle-treated animals.

To further investigate the action of these anthranilates, other inflammatory mediators were also assayed. Thus, paw edema was also induced by prostaglandin E_2_ (PGE2, 3 nmol/paw), compound 48/80 (C48/80, 100 nmol/paw) or 12-*O*-tetradecanoylphorbol-acetate (TPA, 50 pmol/paw). The edema induced by PGE2 and C48/80 was maximal at 30 or 15 min after the injections and reduced in subsequent hours, respectively. All three anthranilates (10 nmol/paw) significantly reduced PGE2-induced paw edema 30 and 60 min-post injection. TPA also increased paw swelling with maximal edema formed 60 minutes after injection. Similarly to what was observed when other phlogistic agents were used, the oedema induced by TPA was also inhibited by ISOAN, MAN and PAN (Fig. 8).

### Effect of anthranilates on nitric oxide production

To further evaluate the effect of anthranilates and to discard the possibility of a reduction in cytokines and NO *in vivo* as a consequence of the reduction in cell migration into the SAP, we decided to perform *in vitro* assays.

We first evaluated a possible direct cytotoxic effect of MAN, ISOAN, and PAN on RAW 264.7 cells. The anthranilates alone, at concentrations up to 100 μM, or in the presence of LPS (1 μg/ml), did not affect cell viability (Data not shown). Therefore, anthranilates were used in concentrations between 1 and 30 μM for the subsequent experiments. The concentration of 100 μM was not used due to a possible effect of the diluent used (DMSO).

**Fig 9:**
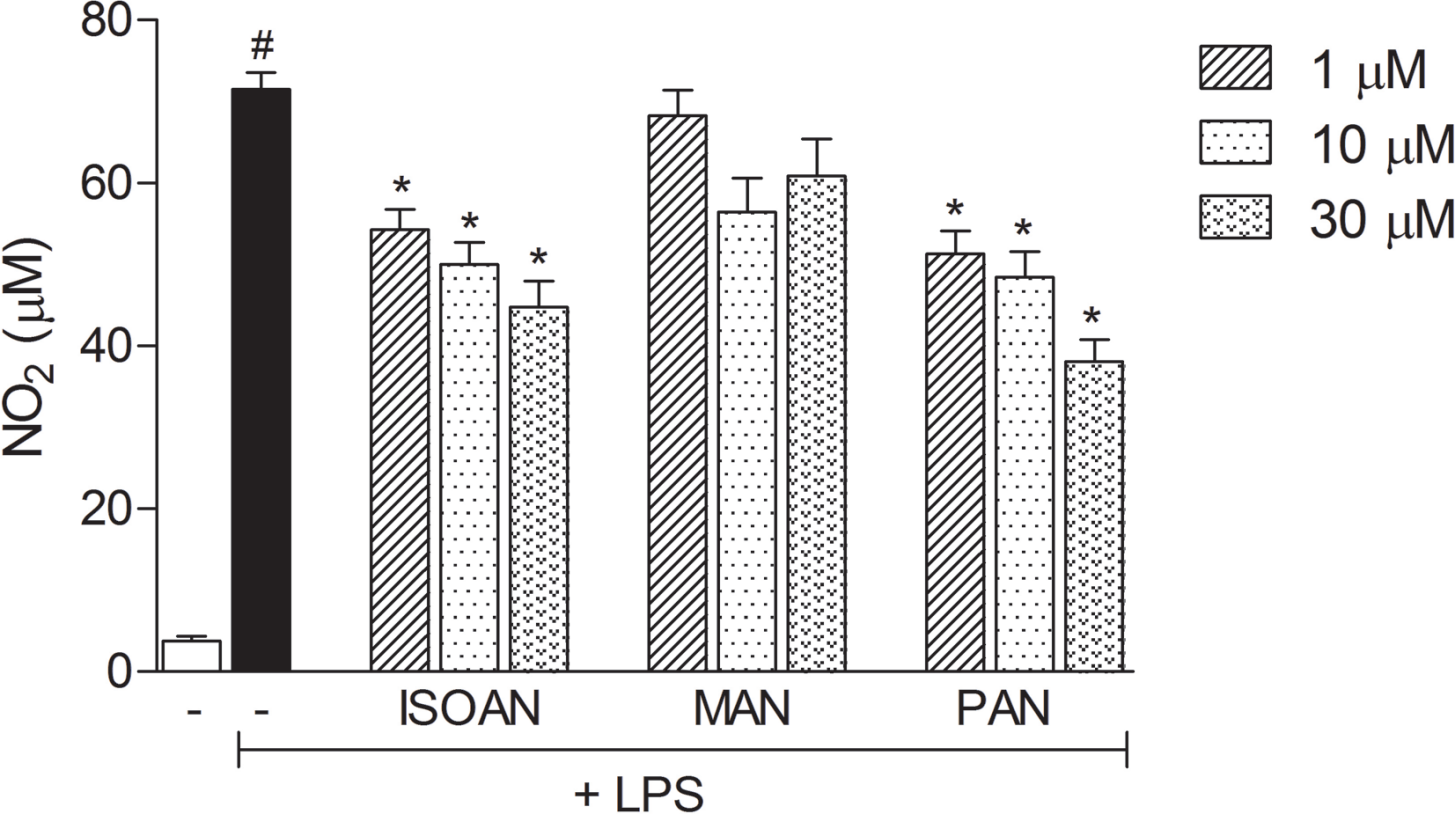
Effects of MAN, PAN or ISOAN on NO production by RAW 264.7 cells. RAW 264.7 cells were either activated or not with LPS (1 μg/ml) and further incubated with anthranilates (1, 10 or 30 μM). After 24 h of incubation the concentration of NO_2_^-^ accumulated in the supernatant was measured by the Griess method. Results are presented as mean ± S.D. (n = 4). Statistical significance was calculated by ANOVA followed by Bonferroni's test. ^#^p<0.05 when comparing the LPS-activated with non-activated cells *p<0.05 comparing LPS-activated cells pre-incubated with anthranilates with the LPS-activated cells.

To assess the effect of anthranilates on LPS-induced NO production in RAW cells, the cells were activated with LPS (1 μg/ml) in the presence or absence of anthranilates (1, 10, 30 μM) and incubated for 24 h. The amount of nitrite, a stable metabolite of NO, was used as the indicator of NO production in the medium. During the 24 h incubation, macrophages in their steady state produced 3.8 ± 1.7 μM NO. In LPS-activated cells, NO production was dramatically increased to 71.5 ± 6.6 μM (Fig. 9). Pretreatment with MAN did not inhibit LPS-induced NO production. PAN caused a concentration-dependent inhibition, corresponding to 24%, 30%, and 37.3% inhibition at 1 μM, 10 μM and 30 μM, respectively and ISOAN reduced NO production by 28.2%, 32.3% and 46.1% at the same doses (Fig. 9).

To rule out the possibility that NO inhibition, shown by anthranilates, is a consequence of it being an NO-scavenger, a cell-free protocol was performed, with the incubation of an NO donor, SNAP, and 100 μM of each anthranilate. None of the anthranilates were able to scavenge NO liberated from SNAP and reduce nitrite levels (data not shown).

## Discussion

In this work we showed that the oral administration of EO obtained from the leaves of *C*. *ternata* reduced some aspects of inflammatory processes. The new compound ternanthranin (ISOAN) from this EO and its synthetic analogues (methyl—MAN and propyl *N*-methylanthranilates—PAN) also demonstrated a significant anti-inflammatory effect.

In a previous study from our group we showed that the oral treatment of mice with EO produced a significant and dose-dependent peripheral and central antinociceptive activity [15]. As the acetic acid-induced writhing reaction is caused by the release of endogenous inflammatory mediators (e.g. histamine, serotonin, bradykinin, cytokines, and eicosanoids) [28], we decide to further investigate the anti-inflammatory profile from EO in two models of inflammation: the licking response induced by formalin and the carrageenan-induced leukocyte migration into the subcutaneous air pouch (SAP). We also tested the isolated substance ternanthranin (ISOAN) and its two analogues MAN and PAN (methyl and propyl *N*-methylanthranilates) in the same models.

The reaction of the animals to an injection of formalin was used to evaluate a possible involvement of neurogenic or inflammatory pain. The formalin response shows two phases. The first phase is characterized by neurogenic pain caused by a direct chemical stimulation of nociceptors. The second phase is characterized by inflammatory pain triggered by a combination of stimuli, including inflammation of the peripheral tissues and mechanisms of central sensitization [29, 30]. In this latter phase, different chemical mediators are involved, as excitatory amino acids, neuropeptides, prostaglandin E2 (PGE2), nitric oxide (NO) and kinins [31–34]. It has been reported that both phases are sensitive to centrally acting drugs such as opioids [35, 36]. However, the second phase is also sensitive to nonsteroidal anti-inflammatory drugs and corticosteroids [20, 37]. In this study our results demonstrated that only the highest dose of EO (30 mg/kg) reduced the time that the animals spent licking the injected paw during the first phase. On the other hand the second phase was inhibited by EO in all tested doses (3, 10 and 30 mg/kg). These results indicate that EO could be acting through inhibition of the formation and/or liberation of the mediators in the paw tissue or by direct blockage of the receptors. These results corroborate the effect of *C*. *ternata* extracts observed in a previous study using the acetic acid-induced contortions model [15].

The anti-inflammatory effect observed in the second phase of the formalin model led us to evaluate EO in the subcutaneous air pouch (SAP) model. This model has been widely used to search for new prototypes of anti-inflammatory drugs [38]. Acute inflammation induced by carrageenan is characterized by accentuated cell migration, predominance of polymorphonuclear cells, increased vascular permeability, extravasation of plasma proteins and increased levels of inflammatory mediators, such as nitric oxide and cytokines [39, 40]. The mechanism of action by which carrageenan induces inflammatory processes seems to be a synergism between several inflammatory mediators, such as bradykinin, serotonin, histamine, prostaglandins and other chemotactic agents [41]. Between the 2^nd^ and the 4^th^ hour after the injection of carrageenan, an intense process of rolling of leukocytes and adhesion of the cells to the endothelial wall is taking place, and during the 6^th^ hour the release of chemotactic agents in the inflammatory site occurs [42]. Although leukocytes have a protective role in inflammation, tissue damage is a deleterious consequence of the intense migration of neutrophils, as observed in immune inflammatory diseases [43].

Our results showed that, in the SAP model, EO reduced the influx of leukocytes, polymorphonuclear and mononuclear cells that migrated to the SAP in response to carrageenan injection. Several studies show that there is a strong association between the inflammatory process and the development of pain and that the inhibition of neutrophil migration reduces the hypernociception induced by different inflammatory stimuli [44, 45]. It is known that inflammatory cells such as neutrophils and macrophages play an important role in inflammatory process by secreting pro-inflammatory mediators, including NO, TNF-α and IL-1β [46, 47]. On the other hand, TNF-α and IL-1β show a relevant effect on neutrophil influx in driving the production of a neutrophil chemoattractant and cell adhesion molecule (e.g. L- and P-selectins) expression at sites of inflammation [48].

Our results confirmed that EO affects chemotaxis through the inhibition of nitric oxide production and pro-inflammatory cytokines levels resulting in the suppression of inflammatory process. It is interesting to note that these inhibitory properties were similar with the anti-inflammatory effects produced by dexamethasone. This steroidal anti-inflammatory drug is effective in inhibiting cell migration, NO production and cytokines levels, as well as leukocytes' influx.

Anti-inflammatory activity of the EO showed in this work could also be related to the high concentrations of terpenoid constituents, such as sabinene, myrcene, terpinen-4-ol, α-terpinene, caryophyllene oxide and phellandrene [15]. These constituents are well known for their numerous biological activities, among them the anti-inflammatory activity [49–52].

Based on the observed effects of EO, we further evaluated the anti-inflammatory effects of ternanthranin, as well as of the two new synthetic analogs, methyl and propyl *N*-methylanthranilates. To discard a possible effect of metabolites of the mentioned anthranilates that could form by a hepatic metabolism of anthranilates administered by oral route, each anthranilate was injected directly into the mice paw before carrageenan or other phlogistic agent administration *i*.*e*., carrageenan, histamine, 5-HT, bradykinin (BK). The inhibitory effect of ternanthranin, methyl and propyl *N*-methylanthranilates in paw edema induced by 5-HT, BK, PGE2 and TPA can be an indication that the anthranilates can act through receptors located in mice paws. These data corroborated those obtained in the carrageenan-induced paw edema. In this model the initial phase of the edema is due to liberation, in the paw, of 5-HT, BK and other mediators [53].

According to the previously observed effects in the formalin model [16] and the effects observed in the paw edema model, we further evaluated the anthranilates in the carrageenan-induced inflammation in the SAP. The inhibitory effect observed in this model could be attributed, at least in part, to receptor blockage in the vascular endothelium. Since, there is a liberation and/or production of several mediators (*i*.*e*., 5-HT, BK, PGE2) occurring in the carrageenan model [53], a possible direct binding of the anthranilates to certain receptors, resulting in the reduction of vasodilation, endothelial cell contraction, protein extravasation, could explain their noted effect in this model. This hypothesis is also backed up by the observation that the anthranilates (injected in mice paws) reduced the edema induced by 5-HT, BK or PGE2. A hypothesis to the observation that anthranilates do not inhibit histamine edema could be that anthranilates do not interact directly with histamine receptors thus do not reducing edema.

Another interesting result was the fact that anthranilates reduced nitric oxide (NO) levels in the SAP cavity. NO is produced by the so called nitric oxide synthases (NOS). The iNOS is an inducible enzyme expressed in inflammatory processes [54, 55]. One of the possible inhibitory mechanism of anthranilates on NO production could be a direct NO-scavenging effect, i.e. a direct reaction with the NO gas in the pouch. This question was discarded by the results of the “cell free system” using an NO donor (SNAP). None of the anthranilates reduced NO liberated from SNAP indicating that there is no direct interaction between the anthranilates and the liberated NO.

Inflammatory cytokines exert an important role in inflammatory process. TNF-α, interferon-γ (IFN-γ) and interleukin 1β (IL-1β) are produced by activated cells resulting in activation of several pathways, increase in vascular permeability and in cell migration and production of chemokines [56]. All anthranilates significantly reduced the levels of produced cytokines in the pouch.

The inhibitory effect of the anthranilates on NO and cytokines production do not seem to be correlated with a cytotoxic effect since an *in vitro* assay (MTT) did not demonstrate a direct effect on macrophages.

In conclusion, in this work we have shown that the EO from the leaves of *C*. *ternata* possesses anti-inflammatory properties. Also, the isolated anthranilate from that EO, ternanthranin, and two of its analogues, methyl and propyl *N*-methylanthranilates, also demonstrated significant anti-inflammatory activity, suggesting them as new candidates for anti-inflammatory drug prototypes.
